# Psychometric Properties of the Measure of Online Disinhibition (MOD) in Chilean Adolescents

**DOI:** 10.3390/bs16030451

**Published:** 2026-03-19

**Authors:** Karina Polanco-Levicán, José Luis Gálvez-Nieto, Ignacio Norambuena-Paredes

**Affiliations:** 1 Facultad de Educación, Universidad Autónoma de Chile, Temuco 4810101, Chile; karina.polanco@cloud.uautonoma.cl; 2 Departamento de Psicología, Universidad Católica de Temuco, Temuco 4780000, Chile; 3 Departamento de Trabajo Social, Universidad de La Frontera, Temuco 4780000, Chile; ignacio.norambuena@ufrontera.cl

**Keywords:** online disinhibition, scale, adolescents, internet, social media

## Abstract

Online disinhibition is a fundamental construct for understanding adolescent behaviour in digital environments. However, in Chile, there are no psychometric studies that support its measurement. In this regard, having valid and reliable tools to assess this phenomenon is key to advancing research on the dynamics of digital interaction and its possible implications for the well-being and online coexistence of adolescents. This study aimed to evaluate the psychometric properties of the Measure of Online Disinhibition (MOD) in a sample of Chilean adolescents. A cross-sectional study was conducted involving 4646 students from 41 secondary education institutions. The sample consisted of 50.2% males, 48.5% females, and 1.4% who identified with another gender category, with an average age of 15.79 years (SD = 1.33). The factorial structure was examined using confirmatory factor analysis, which confirmed the theoretical unidimensional solution. Factorial invariance was examined across gender, internet use, social media use, and age. Scalar invariance was supported for internet use, social media use, and age, while partial scalar invariance was established across gender. Convergent validity was supported by positive, moderate, and statistically significant correlations with the Global Assessment of Internet Trolling (GAIT). Finally, the scale demonstrated adequate internal consistency, supporting its use in the Chilean adolescent population.

## 1. Introduction

### 1.1. Digital Communication and Online Behaviour in Adolescence

Digital communication has expanded significantly among adolescents as more regions gain access to internet connectivity (Nagata et al., 2025; Salza & Samuel, 2025; Stuart & Scott, 2021). The virtual context has become an attractive space for adolescents in this hybrid world, enabling socialisation, entertainment, and multiple online activities (Heinrich et al., 2025; Neira et al., 2025; van der Wal et al., 2024). However, adolescents are also exposed to inappropriate content and harmful interactions (Ziegel et al., 2025). The interest in staying connected, together with lower critical thinking development and low risk perception regarding social media and the internet, may increase exposure to adverse experiences (Neira et al., 2025; Sandoval et al., 2025; Torrubia-Pérez et al., 2025). In this context, greater online disinhibition has been linked to aggressive behaviours affecting adolescents’ mental health (Polanco-Levicán et al., 2025; Weisskirch, 2025).

The concept of online disinhibition refers to the reduction in the limitations or restrictions that people apply to their actions on the internet compared to the physical context. This idea is based on the notion that perceptions and experiences change in virtual interactions, influencing how individuals behave, think, and feel (Stuart & Scott, 2021). Internet interactions differ from face-to-face interactions due to anonymity and invisibility (R. A. Scott et al., 2022a). In this context, individuals show fewer behavioural restrictions than in interpersonal relationships in physical settings, as the characteristics of online interactions influence the interpretation of personal and social responsibility (Stuart & Scott, 2021; Suler, 2004).

Suler (2004) proposed that online disinhibition manifests through six characteristics that facilitate this effect that occurs in people when they participate in virtual contexts; these are: anonymity, invisibility, asynchronicity, solipsistic introjection, dissociative imagination, and minimisation of authority. The author (Suler, 2004) also states that these conditions promote disinhibited behaviour, which can appear in two forms: the first is a benign form, as it involves greater self-expression, emotional openness, and the search for social support, therefore the outcomes for both the person and others are positive; and the second is a toxic disinhibition, which involves negative and harmful behaviours toward other people.

Various studies have shown associations between online disinhibition and constructs such as moral disengagement and cyber aggression (Tan, 2024; Yang et al., 2021). Specifically, online disinhibition is linked to cyberbullying, which significantly affects adolescents’ mental health worldwide (Charoenwanit et al., 2025; L. Wang et al., 2024; X. Wang et al., 2022). Evidence indicates that online disinhibition moderates the relationship between revenge motivation and cyberbullying perpetration (Ding et al., 2025). Recent empirical evidence further shows that online disinhibition not only relates to cyberbullying perpetration but also moderates the victim–perpetrator cycle. The relationship between victimisation and perpetration is stronger among individuals with high levels of online disinhibition, thereby intensifying the transition from victimization to an aggressor role in virtual contexts (Kasturiratna & Hartanto, 2025). Additionally, online disinhibition mediates the relationship between moral disengagement and cyberbullying by fostering an environment in which adolescents feel less constrained by social norms or personal consequences, facilitating the justification and minimisation of harm toward others (L. Wang et al., 2024). Online disinhibition is positively associated with victimisation through technology-facilitated sexual violence, particularly among youth from sexual and gender minorities (Amadori & Brighi, 2025).

A specific relationship exists between trolling—as a form of digital violence—and online disinhibition. In a sample of Chinese adolescents, evidence shows that online disinhibition moderates the relationship between parental phubbing and children’s trolling behaviour, with this association becoming stronger as online disinhibition increases (Liao et al., 2024). Likewise, the mediated relationship between trolling victimisation and reactive trolling through revenge motivation is stronger among individuals with high online disinhibition (Mao & Hu, 2025). Furthermore, greater online disinhibition is associated with a stronger relationship between supportive trolling and overt trolling (Montez & Kim, 2025). Digital anonymity is also relevant for understanding online behaviours, as both anonymity and the sense of collectivity generated online influence trolling behaviour through a diffuse sense of responsibility (Hossain et al., 2025). Thus, the perception of individual and group anonymity influences the intention to engage in collective trolling, with the level of online disinhibition playing a relevant role (Li et al., 2022).

It is important to contextualise that the stage of adolescence is characterised by an interest in interacting and belonging to the peer group, as well as by the search for acceptance and approval. Considering technological development, the possibility of socialising is always present and in all places within the virtual context (Pérez-Torres, 2024). Thus, adolescents may increase their online participation to strengthen friendship bonds (Angelini et al., 2024), thereby promoting a more active online engagement (Stevic, 2024). Along the same lines, it allows adolescents to participate in various activities associated with exploration, thereby reinforcing aspects of identity development (Avci et al., 2025). Adolescents also tend to seek greater autonomy, which may hinder parental supervision of their online behaviours, although parents may use different strategies to monitor their children’s behaviour in virtual environments (Hernandez et al., 2024; Pérez-Torres, 2024).

In the same vein, the importance of using mobile phones to stay on social media is positively related to the relevance of staying connected to generate social ties, as this is associated with greater closeness in friendships. In this regard, differences can be observed, showing that adolescent girls, when valuing technology as a means of connection, use their mobile phones for more extended periods than boys, who also use technology for other reasons (Al-Jbouri et al., 2024). These findings demonstrate that digital technology and social media not only serve a communicative purpose but also function as a key relational space in the construction and maintenance of friendship bonds during adolescence.

### 1.2. Measurement of Online Desinhibition and Previous Research

Stuart and Scott (2021) propose a theoretical and methodological approach to improve the understanding and assessment of this construct across different age groups. They state that online disinhibition can be understood as a behavioural tendency in virtual contexts that does not necessarily take on a negative or positive character, but rather reflects personal differences expressed through interactions with online features (Stuart & Scott, 2021). Consequently, they suggest the possibility of assessing online disinhibition without considering behavioural outcomes. A strength of this scale is that it specifically evaluates online disinhibition without assessing characteristics of online environments or experiences (antecedents or outcomes) related to disinhibition.

To evaluate this construct, Stuart and Scott (2021) presented the Measure of Online Disinhibition (MOD). This scale was applied to a sample of U.S. adults aged 18 to 70. It presents a unidimensional factorial structure with 12 items and reports adequate validity and reliability indices (Cronbach’s alpha = 0.95). This scale was adapted and validated in Chilean university students with an average age of 20.57 years. The results showed that the unidimensional factorial structure was maintained and that it had adequate psychometric properties (Polanco-Levicán & Galvez-Nieto, 2024). Another study conducted with university students in Turkey reported adequate levels of reliability and validity indices in this population (Celik et al., 2025; Gunnoo et al., 2025).

In a complementary manner, Syrjämäki et al. (2024) used the scale in Finland, reporting outstanding reliability (Cronbach’s alpha = 0.92) and showing that online disinhibition mediates the relationship between difficulties in emotional regulation and uncivil communication. Likewise, Gunnoo et al. (2025) found in the field of digital criminology that the MOD, together with the use of dating applications and dark triad traits, predicts the perpetration of technology-facilitated sexual violence. In Japan, Wen and Miura (2024) proposed a multidimensional version (MMOD) with adequate concurrent validity relative to the MOD, while Schmitt et al. (2025) demonstrated that although the original measure captures the phenomenon neutrally, the toxic dimension explains deviant behaviours in digital environments more accurately.

The scale has been used in several studies. In the study by Stuart and Scott (2021), an increase in connection time was associated with greater disinhibition in digital interactions, and higher levels of online disinhibition were associated with lower perceived well-being. According to R. A. Scott et al. (2022b), the perception of protection (perceived protection, invisibility, and safety) among young adults increases disinhibition in their online behaviour. In the same sample of participants aged 17 to 25 years, age did not show a significant association with online disinhibition. In this sense, the construct proposed by the authors (Stuart & Scott, 2021) will facilitate understanding and differentiation between digital experiences and face-to-face interactions.

### 1.3. Research Gap, Chilean Context and Aim of the Study

Despite the growing use of the Measure of Online Disinhibition (MOD) in different countries and populations, evidence regarding its psychometric performance in adolescent samples remains limited. In Chile, the scale has been adapted and validated only in university students, leaving a gap in the assessment of this construct among adolescents. Therefore, it is essential to evaluate instruments with appropriate validity and reliability indices for this population, particularly for constructs such as online disinhibition, which are linked to various forms of virtual aggression (Ziegel et al., 2025). As noted by Stuart and Scott (2021), work must continue with people from different age groups in various parts of the world.

Understanding the use of digital devices is considered essential for protecting adolescents’ well-being (Ives et al., 2025). Thus, schools can intervene appropriately, both preventively and reactively, in response to problems that arise in online contexts. According to the 2024 Census (Instituto Nacional de Estadísticas, 2025), 93.2% of households in Chile have Internet access. In this context of high connectivity, 79% of students enrolled between 7th grade and 12th grade access the Internet from their homes (Kids Online, 2022). Within the school setting, 58% of institutions have Internet connectivity in virtually all their facilities and 27% in most areas; moreover, teachers frequently assign activities that involve individual and collaborative Internet use. At the individual level, 87% of students report owning a personal mobile phone with Internet access, and the average age of obtaining a first mobile phone has decreased to 8.9 years (Kids Online, 2022). Additionally, another study indicates that all participating secondary school students had at least one social media profile (Gómez-Urrutia & Jiménez Figueroa, 2022).

Therefore, educational institutions and caregivers must become involved and understand their students’ online experiences. At the same time, it is vital to develop public policies that promote appropriate online relationships and reduce the harm caused by these interactions (Ziegel et al., 2025).

Consequently, two hypotheses were established in this study: first, that the Measure of Online Disinhibition (MOD) would present a unidimensional structure and that reliability levels in the Chilean context would be adequate; second, it was proposed that the scale scores would remain stable up to the level of scalar invariance, with such invariance evaluated according to gender, internet use, social media use and age. Accordingly, this study aimed to evaluate the psychometric properties of the Measure of Online Disinhibition (MOD) in a sample of Chilean adolescents.

## 2. Materials and Methods

### 2.1. Participants

The study considered a population of 4646 students from secondary education institutions in Chile, belonging to 41 educational establishments. As shown in Table 1, the sample consisted of 50.2% men, 48.5% women, and 1.4% who identified with another gender category, with a mean age of 15.79 years (SD = 1.33). Likewise, regarding the time spent online, 43.2% of students reported being connected for 1–4 h per day for leisure, while 55.2% indicated spending 5 h or more. A total of 1.6% reported not using the internet for recreational purposes.

Regarding time spent on social media, 57.2% of students reported using it for 1–4 h per day, while 41% reported using it for 5 h or more. Meanwhile, 1.8% indicated not using social media. With respect to family background, most students (84.3%) come from urban contexts, while 15.7% report coming from rural areas.

### 2.2. Instruments

To achieve the study’s objectives, a sociodemographic questionnaire was administered to collect relevant information about the students. This instrument made it possible to obtain background information on gender, age, educational level, and family origin, as well as characteristics related to school type, ethnic background, and patterns of internet and social media use.

*Online Disinhibition Scale (Measure of Online Disinhibition, MOD)*. The MOD scale is an instrument that measures the experience of reduced restraint while online (Stuart & Scott, 2021). This instrument is a self-report scale composed of 12 items answered using a five-point ordinal scale, 1 = not at all like me, 5 = very much like me, (see Supplementary Table S1). Psychometric studies have shown that the MOD exhibits a one-factor structure. Regarding reliability and validity indices, the scale demonstrated adequate construct validity for a single dimension and significant correlations with other constructs, including trolling, time online, and toxic and benign disinhibition. In terms of reliability, it showed adequate internal consistency, with a Cronbach’s alpha of 0.97 (Stuart & Scott, 2021). This scale also underwent a psychometric study with university students, demonstrating adequate levels of validity and reliability (Polanco-Levicán & Galvez-Nieto, 2024).

Additionally, the *Global Assessment of Internet Trolling Scale* (GAIT) (Buckels et al., 2014) is a scale designed to assess behaviours associated with trolling in digital environments (Craker & March, 2016; Liu et al., 2022). The scale consists of 4 items, each rated on a 5-point ordinal scale (1 = strongly disagree to 5 = strongly agree). Regarding its reliability, the study by Liu et al. (2022) reported a Cronbach’s alpha coefficient of α = 0.74, which is acceptable for research use. With respect to construct validity, the confirmatory factor analysis indicated a good fit of the one-factor model to the data (RMSEA = 0.035; SRMR = 0.011; TLI = 0.99; CFI = 0.99), supporting the theoretical structure proposed by the original authors.

### 2.3. Procedure

The Chilean version of the Measure of Online Disinhibition (MOD), previously adapted and validated in Chilean university students (Polanco-Levicán & Galvez-Nieto, 2024), was used. According to Polanco-Levicán and Galvez-Nieto (2024), the initial adaptation was conducted in accordance with the guidelines of the International Test Commission (2017). In the present study, the items were reviewed by a panel of experts to ensure their linguistic and conceptual appropriateness for an adolescent population. A pilot administration was subsequently conducted with adolescents to evaluate the clarity and relevance of the content. The findings indicated that the items were comprehensible; therefore, no further modifications were necessary.

Prior to administration, coordination was carried out with the administrative and teaching staff of the participating educational institutions to schedule the administration of the instruments during regular school hours. Data collection was conducted in classroom settings through group administration using the digital platform QuestionPro, ensuring standardized delivery of the questionnaire. The process was supervised by previously trained members of the research team, who provided uniform instructions and addressed procedural questions without influencing participants’ responses.

Before administering the instruments, the principals of the participating educational institutions were contacted, and they formally authorized access to the sample. Subsequently, informed consent was requested from parents or legal guardians, and after their approval, the students provided their informed assent to participate.

Participation was voluntary and based on informed consent and student assent, in accordance with international ethical principles for research. Strict anonymity and confidentiality were maintained; the questionnaires did not include personally identifying data, were coded solely for analytical purposes, and students were informed of their right to withdraw from the study at any time without academic consequences. The entire process was conducted under the direct supervision of the responsible research team.

The entire procedure, along with the corresponding ethical considerations, was reviewed and approved by the Ethics Committee of the Universidad de La Frontera, Chile.

Regarding missing data, the platform QuestionPro (QuestionPro Inc., Austin, TX, USA) was configured with control filters that required participants to complete each question before proceeding, thereby reducing the likelihood of omissions. Nevertheless, following data download and cleaning, records containing incomplete responses were excluded from the analysis, and only cases with complete information across all study variables were retained. This procedure allowed the use of a final complete sample, ensuring the consistency of the psychometric analyses and the stability of the estimates.

### 2.4. Data Analysis

First, descriptive analyses were conducted for the MOD scale items, including mean, standard deviation, skewness, kurtosis, and normality tests, using SPSS v.25. Confirmatory factor analyses (CFA) were performed using Mplus v8.1 (Muthén & Muthén, 2017), employing the maximum likelihood estimation with robust standard errors (MLR) method. This estimator is particularly recommended when multivariate normality is absent, as it adjusts standard errors and provides more robust estimates.

The quality of model fit was evaluated using values ≥ 0.90 for the Comparative Fit Index (CFI) and the Tucker–Lewis Index (TLI) as adequate indicators, and values ≤ 0.08 for the Root Mean Square Error of Approximation (RMSEA). Likewise, factorial invariance was assessed according to Vandenberg and Lance’s (2000) proposal, by successively comparing the following models: M0 (configural, equality in the number of factors), M1 (metric, equality in factor loadings), and M2 (scalar, equality in intercepts).

Convergent validity was estimated using Pearson correlations (r), following the interpretation criteria suggested by Cohen (2013) and Morales et al. (2003). The evaluation of factorial invariance was conducted considering the recommended cut-off points: ΔTLI = 0 as a perfect indicator and ≤0.01 as acceptable, as well as ΔRMSEA ≤ 0.015, which constitutes evidence of measurement invariance.

Finally, the reliability of the scales was calculated using JASP v.0.19, applying different internal consistency coefficients: McDonald’s omega (ω), Greatest Lower Bound (GLB), and Cronbach’s alpha (α), in accordance with the criteria proposed by Trizano-Hermosilla et al. (2021).

## 3. Results

### 3.1. Data Analysis

Table 2 presents the descriptive statistics for the 12 MOD items. As shown, item 9 obtained the highest mean (M = 2.71; SD = 1.28), followed by item 4 (M = 2.70; SD = 1.17). In contrast, item 2 showed the lowest mean (M = 2.05; SD = 1.08), followed by item 11 (M = 2.37; SD = 1.27). Regarding distribution characteristics, skewness values ranged from 0.12 (item 4) to 0.91 (item 2), indicating slight positive skewness in most items, which implies that scores tend to cluster toward the lower end of the scale. Kurtosis values were predominantly negative, ranging from −1.16 (item 5) to 0.07 (item 2), suggesting lighter-tailed distributions. The Kolmogorov–Smirnov test values (0.16–0.24) show deviations from expected normality, which is consistent with ordinal data and supports the use of robust estimators in subsequent analyses.

### 3.2. Confirmatory Factor Analysis

To evaluate the factorial structure of the MOD, a confirmatory factor analysis was conducted (Table 3). The results adequately support the unidimensional structure of the instrument, showing satisfactory fit indices: MLR-χ^2^(54) = 1021.512, *p* < 0.001, CFI = 0.929, TLI = 0.913, RMSEA = 0.062 (90% CI 0.059–0.066), SRMR = 0.038.

### 3.3. Factorial Invariance

Measurement invariance was examined across gender, Internet use, social media use, and age. As shown in Table 4, configural invariance was supported across all groups, indicating that the factorial structure was equivalent across the different categories analyzed. Regarding gender, the metric model demonstrated an adequate fit compared to the configural model, supporting the invariance of factor loadings. However, the full scalar model did not meet the recommended criteria, indicating a lack of complete scalar invariance between males and females. Therefore, a partial scalar invariance model was estimated by freeing the intercept of item 5, which resulted in an acceptable model fit and allowed for meaningful comparisons of latent means across gender. In contrast, both metric and scalar invariance were supported for Internet use, social media use, and age, as changes in CFI and RMSEA remained below conventional thresholds. Overall, these findings provide evidence of full measurement invariance across levels of Internet use, social media use, and age, and partial scalar invariance across gender.

### 3.4. Convergent Validity

To evaluate the convergent criterion validity of the online disinhibition measure (MOD), bivariate correlations were analysed with a theoretically related behaviour: trolling. The results show a positive, statistically significant correlation between online disinhibition and trolling (r = 0.395, *p* < 0.001). This magnitude corresponds to a moderate effect, indicating that higher levels of online disinhibition are associated with a greater tendency to engage in trolling behaviours.

### 3.5. Internal Consistency Reliability

The online disinhibition measure (MOD) demonstrated adequate internal consistency (Table 5). The coefficients ranged from 0.880 for Cronbach’s alpha to 0.911 for the GLB. In addition, the 95% confidence intervals were narrow, supporting the precision and stability of the estimates.

## 4. Discussion

This study aimed to evaluate the psychometric properties of the Measure of Online Disinhibition (MOD) in a sample of Chilean adolescents. The findings of this study provide solid empirical evidence of the MOD scale’s validity in this population, confirming its unidimensional factorial structure. The factorial structure identified in this study is consistent with the original proposal by Stuart and Scott (2021), who developed the scale to assess the experience of reduced constraints in online contexts—that is, the tendency of individuals to act, think, or express themselves differently in virtual environments compared to face-to-face interactions (Barlett & Scott, 2023; J. E. Scott & Barlett, 2023; R. A. Scott et al., 2022b).

Regarding the evaluation of factorial invariance, the results indicate that configural invariance was supported across gender, Internet use, social media use, and age, suggesting that the construct maintains an equivalent factorial structure across the groups analysed. Metric invariance was also supported for all grouping variables, indicating that factor loadings are comparable across gender, levels of Internet use, social media use, and age.

With respect to scalar invariance, full scalar invariance was achieved for Internet use, social media use, and age, as reflected by minimal changes in nested fit indices. However, for gender, full scalar invariance was not supported. A partial scalar invariance model was therefore established by freeing the intercept of one item, resulting in an acceptable model fit. Overall, these findings indicate that the MOD operates equivalently across levels of digital exposure and age, and that meaningful latent mean comparisons can also be conducted across gender under partial scalar invariance, supporting its robustness for group comparisons.

The evaluation of the MOD’s convergent validity is supported by its association with conceptually related behaviours, such as trolling (Moor & Anderson, 2019; Tian et al., 2024; Voggeser et al., 2018). This link confirms that the scale captures psychological dispositions that facilitate the expression of disruptive behaviours in virtual environments, with evidence positioning online disinhibition as an explanatory factor for various forms of digital aggression and participation in antisocial interactions (Giannakopoulos & Prassou, 2025; Hudon et al., 2025; Nagar et al., 2025).

Taken together, the reliability evidence confirms that the MOD exhibits robust internal consistency among Chilean adolescents, aligning with international findings of psychometric stability across diverse cultural contexts (Stuart & Scott, 2021; Celik et al., 2025). These results reinforce its usefulness as a valid measure for understanding online disinhibition processes during adolescence, a critical stage in which digital interaction shapes identities and social bonds (Gómez-Urrutia & Jiménez Figueroa, 2022; Márquez et al., 2023; Roberts & David, 2023).

The strength of these findings indicates that the MOD is sensitive to capturing individual differences in online disinhibition and offers a reliable empirical framework for exploring its role in associated phenomena, such as cyberbullying, trolling, and other forms of risky digital interaction (Aledeh et al., 2024; Ray et al., 2024). Thus, the scale positions itself as a relevant tool for comparative research and cross-cultural studies aimed at understanding the impact of behaviour in virtual environments (Stuart & Scott, 2021).

These results underscore the importance of instruments that enable the assessment of emerging constructs in adolescents’ digital lives. From a theoretical perspective, the findings contribute to strengthening the construct of online disinhibition within the field of digital psychology, particularly by situating it within a developmental stage characterized by processes of behavioral regulation and social interaction in virtual contexts distinct from face-to-face interaction. The evidence obtained supports its integration into explanatory frameworks that examine the interplay between the structural features of the virtual environment and developmental processes specific to adolescence, thereby reinforcing its contribution to understanding technology-mediated behavior.

From an applied perspective, the availability of a psychometrically robust measure in an adolescent population allows this construct to be incorporated into strategies aimed at the promotion, prevention, and remediation of digital well-being, particularly through its inclusion in institutional assessments and in the design of digital interaction management strategies. This facilitates the planning and implementation of educational programs focused on developing competencies for interaction in virtual environments and on the responsible use of technology, fostering greater awareness of the dynamics inherent to these contexts. It also provides empirical evidence to inform the development of educational guidelines and regulatory frameworks that explicitly consider the psychological factors involved in online interaction during adolescence, supporting targeted interventions and the promotion of safe and responsible digital interaction.

### Limitations and Future Research

A relevant limitation of the present study is its cross-sectional design, which prevents establishing causal relationships between online disinhibition and other psychosocial variables. Therefore, future research should adopt longitudinal designs to examine the temporal stability of the construct and its role in shaping adolescents’ digital behaviors.

Another important line of research involves examining the role of cultural factors in the expression of online disinhibition. Although the scale has demonstrated adequate psychometric performance in different countries (Celik et al., 2025; Stuart & Scott, 2021), social norms and values may influence how this phenomenon manifests itself (Mueller-Coyne et al., 2022; Tennakoon et al., 2024). Cross-cultural studies will help determine the extent to which the construct remains stable across diverse sociocultural contexts (Cheung et al., 2021; Polanco-Levicán & Galvez-Nieto, 2024; X. Wang et al., 2022).

Finally, further research should be conducted to examine the associations between online disinhibition and relevant psychosocial variables in order to strengthen the evidence for its external validity. Although previous studies have linked this concept to behaviors such as trolling and cyberbullying (Barlett, 2023; Barlett et al., 2020; L. Wang et al., 2024), incorporating variables related to adolescent well-being (Norambuena-Paredes et al., 2025), such as emotional regulation or digital empathy, may contribute to a more comprehensive understanding of its implications in digital environments.

## 5. Conclusions

The findings of this study provide robust evidence of the psychometric properties of the Measure of Online Disinhibition (MOD) in Chilean adolescents, confirming its unidimensional structure and adequate reliability. These results are consistent with previous international research that reinforces the usefulness of the scale as a valid and reliable measure of online disinhibition during a developmental stage susceptible to digital technologies.

The consistency of the results indicates that the MOD accurately captures individual variations in the degree of disinhibition in virtual contexts and constitutes a solid tool for analysing associated phenomena such as trolling and cyberbullying. The observed correlations with these behaviours confirm the instrument’s relevance for studying digital interaction dynamics with direct implications for school coexistence and adolescent mental health.

Beyond its psychometric contribution, this study highlights the need to deepen the understanding of online disinhibition as a construct that goes beyond the dichotomy between adaptive and maladaptive expressions. The available evidence indicates that this disposition can foster both risky behaviours and prosocial manifestations in the digital environment, which warrants consideration as a transversal and complex factor in research on technology-mediated interactions.

In applied terms, the availability of an instrument validated in a Chilean adolescent population opens the possibility of designing preventive interventions and evidence-based digital literacy programs. Likewise, its use in international comparative studies will help advance to a broader, more nuanced understanding of the impact of online disinhibition on young people’s lives, contributing to the design of public policies aimed at promoting virtual environments that are safer, more critical, and healthier.

## Figures and Tables

**Table 1:** Main Characteristics of the Sample.

Variables	Categories	n (%)
Gender	Male	50.2%
	Female	48.5%
	Other	1.4%
Internet Use	More than 5 h per day	55.2%
	Between 1 and 4 h per day	43.2%
	Never	1.6%
Social Media Use	More than 5 h per day	41%
	Between 1 and 4 h per day	57.2%
	Never	1.8%
Area of Residence	Urban	84.3%
	Rural	15.7%

**Table 2:** Descriptive statistics.

Items	M	Sd	g1	g2	K-S Test
It1	2.521	1.271	0.402	−0.960	0.202
It2	2.050	1.080	0.910	0.074	0.244
It3	2.532	1.241	0.360	−0.983	0.205
It4	2.700	1.171	0.122	−0.895	0.163
It5	2.694	1.371	0.300	−1.161	0.195
It6	2.631	1.311	0.298	−1.091	0.190
It7	2.610	1.250	0.300	−0.961	0.191
It8	2.592	1.213	0.280	−0.921	0.191
It9	2.712	1.281	0.201	−1.100	0.191
It10	2.463	1.250	0.484	−0.800	0.210
It11	2.370	1.267	0.603	−0.741	0.221
It12	2.500	1.170	0.331	−0.801	0.195

Note: M = Mean; SD = Standard deviation; g1 = Skewness; g2 = Kurtosis; K–S = Kolmogorov–Smirnov.

**Table 3:** Standardized estimates for the MOD One-Factor model.

Item	λ (STDYX)	SE	*p*	Residual Variance (θ)	R^2^
It1	0.618	0.012	<0.001	0.618	0.382
It2	0.546	0.012	<0.001	0.701	0.299
It3	0.488	0.014	<0.001	0.762	0.238
It4	0.581	0.012	<0.001	0.662	0.338
It5	0.545	0.012	<0.001	0.703	0.297
It6	0.699	0.010	<0.001	0.512	0.488
It7	0.669	0.011	<0.001	0.553	0.447
It8	0.660	0.011	<0.001	0.565	0.435
It9	0.687	0.010	<0.001	0.529	0.471
It10	0.618	0.012	<0.001	0.618	0.382
It11	0.642	0.011	<0.001	0.588	0.412
It12	0.638	0.011	<0.001	0.593	0.407

Note: λ = standardized factor loading (STDYX); SE = standard error; θ = standardized residual variance; R^2^ = squared multiple correlation. All loadings were statistically significant (*p* < 0.001). Residual variances correspond to 1 − R^2^.

**Table 4:** Evaluation of measurement invariance across groups.

Group	Model	MLR-χ^2^ (df)	RMSEA	CFI	TLI	SRMR	ΔRMSEA	ΔCFI	Decision
Gender	Configural	1039.886 * (108)	0.062	0.929	0.913	0.039			Accepted
	Metric invariance	1086.093 * (119)	0.060	0.926	0.918	0.040	−0.002	−0.003	Accepted
	Scalar invariance	1464.545 * (130)	0.068	0.898	0.896	0.049	0.008	−0.028	Rejected
	Scalar partial invariance (It5 intercept freed)	1334.876 (131 *)	0.064	0.907	0.906	0.074	0.004	−0.019	Accepted (partial)
Internet Use	Configural	1087.367 * (108)	0.063	0.926	0.910	0.039			Accepted
	Metric invariance	1146.323 * (119)	0.061	0.923	0.914	0.043	−0.002	−0.003	Accepted
	Scalar invariance	1186.150 * (130)	0.059	0.920	0.919	0.042	−0.002	−0.003	Accepted
Social Media Use	Configural	1068.503 * (108)	0.062	0.929	0.913	0.039			Accepted
	Metric invariance	1115.949 * (119)	0.060	0.926	0.918	0.041	−0.002	−0.003	Accepted
	Scalar invariance	1155.250 * (130)	0.058	0.924	0.923	0.041	−0.002	−0.002	Accepted
Age	Configural	1125.782 * (108)	0.064	0.926	0.910	0.039	—	—	Accepted
	Metric invariance	1171.434 * (119)	0.062	0.924	0.915	0.041	−0.002	−0.002	Accepted
	Scalar invariance	1213.457 * (130)	0.060	0.921	0.920	0.041	−0.002	−0.003	Accepted

Note: MLR = maximum likelihood estimation with robust standard errors; χ^2^ = chi-squared; df = degrees of freedom; RMSEA = root mean square error of approximation; CFI = comparative fit index; TLI = Tucker–Lewis index; SRMR = standardised root mean square residual; ΔRMSEA = change in RMSEA between nested models; ΔCFI = change in CFI between nested models. * *p* < 0.001.

**Table 5:** Reliability.

Estimate	McDonald’s ω	Cronbach’s α	Greatest Lower Bound
Point estimate	0.881	0.880	0.911
95% CI lower bound	0.876	0.875	0.907
95% CI upper bound	0.886	0.885	0.917

## Data Availability

The dataset for the study is available from the corresponding author upon reasonable request due to ethical restrictions.

## Supplementary Materials

The following supporting information can be downloaded at: https://www.mdpi.com/article/10.3390/bs16030451/s1, Table S1. Items in the original Spanish version of the MOD scale and an English version of the instrument.

## References

[B1-behavsci-16-00451] Aledeh M., Sokan-Adeaga A. A., Adam H., Aledeh S., Kotera Y. (2024). Suggesting self-compassion training in schools to stop cyberbullying: A narrative review. Discover Psychology.

[B2-behavsci-16-00451] Al-Jbouri E., Volk A. A., Spadafora N., Andrews N. C. (2024). Friends, followers, peers, and posts: Adolescents’ in-person and online friendship networks and social media use influences on friendship closeness via the importance of technology for social connection. Frontiers in Developmental Psychology.

[B3-behavsci-16-00451] Amadori A., Brighi A. (2025). Technology-facilitated sexual violence among sexual and gender minority youth: The moderating role of digital resilience. Computers in Human Behavior.

[B4-behavsci-16-00451] Angelini F., Gini G., Marino C., Van Den Eijnden R. (2024). Social media features, perceived group norms, and adolescents’ active social media use matter for perceived friendship quality. Frontiers in Psychology.

[B5-behavsci-16-00451] Avci H., Baams L., Kretschmer T. (2025). A systematic review of social media use and adolescent identity development. Adolescent Research Review.

[B6-behavsci-16-00451] Barlett C. P. (2023). Cyberbullying as a learned behavior: Theoretical and applied implications. Children.

[B7-behavsci-16-00451] Barlett C. P., Heath J. B., Madison C. S., DeWitt C. C., Kirkpatrick S. M. (2020). You’re not anonymous online: The development and validation of a new cyberbullying intervention curriculum. Psychology of Popular Media.

[B8-behavsci-16-00451] Barlett C. P., Scott J. E. (2023). Racism behind the screen: Examining the mediating and moderating relationships between anonymity, online disinhibition, and cyber-racism. Journal of Personality and Social Psychology.

[B9-behavsci-16-00451] Buckels E. E., Trapnell P. D., Paulhus D. L. (2014). Trolls just want to have fun. Personality and Individual Differences.

[B10-behavsci-16-00451] Celik F., Savci M., Sahin Y., Griffiths M. D., Kose N. (2025). Validity and reliability study of the Turkish version of the measure of online disinhibition (MOD). Journal of History School.

[B11-behavsci-16-00451] Charoenwanit S., Nintachan P., Sangon S., Orathai P. (2025). The moderating effect of self-esteem on the relationships between associated factors and cyberbullying behavior among Thai adolescents. Psychology in the Schools.

[B12-behavsci-16-00451] Cheung C. M., Wong R. Y. M., Chan T. K. (2021). Online disinhibition: Conceptualization, measurement, and implications for online deviant behavior. Industrial Management & Data Systems.

[B13-behavsci-16-00451] Cohen J. (2013). Statistical power analysis for the behavioral sciences.

[B14-behavsci-16-00451] Craker N., March E. (2016). The dark side of Facebook^®^: The dark tetrad, negative social potency, and trolling behaviours. Personality and Individual Differences.

[B15-behavsci-16-00451] Ding J., Lin Y., Chen I.-H. (2025). Why individuals with trait anger and revenge motivation are more likely to engage in cyberbullying perpetration? The online disinhibition effect. Frontiers in Public Health.

[B16-behavsci-16-00451] Giannakopoulos G., Prassou A. (2025). Mediating and moderating mechanisms in the relationship between social media use and adolescent aggression: A scoping review of quantitative evidence. European Journal of Investigation in Health, Psychology and Education.

[B17-behavsci-16-00451] Gómez-Urrutia V., Jiménez Figueroa A. (2022). Identity in the digital age: Elaboration of social media profiles in Chilean teenagers. Convergencia.

[B18-behavsci-16-00451] Gunnoo A., Jackson M., Saling L. L. (2025). The dark triad, dating app use and online disinhibition positively predict technology-facilitated sexual violence perpetration. Journal of Criminology.

[B19-behavsci-16-00451] Heinrich A. J., Heitmayer M., Smith E., Zhang Y. (2025). Experiencing hybrid spaces: A scoping literature review of empirical studies on human experiences in cyber-physical environments. Computers in Human Behavior.

[B20-behavsci-16-00451] Hernandez J. M., Ben-Joseph E. P., Reich S., Charmaraman L. (2024). Parental monitoring of early adolescent social technology use in the US: A mixed-method study. Journal of Child and Family Studies.

[B21-behavsci-16-00451] Hossain M. A., Quaddus M., Akter S., Mikalef P., Warren M. (2025). Trolling in social media: A deindividuation and contagion perspective. Information & Management.

[B22-behavsci-16-00451] Hudon A., Harvey E., Nicolas S., Dufour M., Guérin-Thériault C., Bérubé-Fortin J., Combey I., Yue Y. C., Perreault A., Borduas Pagé S., MacDermott V. (2025). Cyberpsychopathy: A multidimensional framework for understanding psychopathic traits in digital environments. European Journal of Investigation in Health, Psychology and Education.

[B23-behavsci-16-00451] Instituto Nacional de Estadísticas (2025). Censo 2024: El 61,1% de los hogares residen en una vivienda propia y el 26,2% en una vivienda arrendada.

[B24-behavsci-16-00451] International Test Commission (2017). The ITC guidelines for translating and adapting test.

[B25-behavsci-16-00451] Ives L. S. E., Patón A. H., Buratti M. A. F., Pitti J. Á., Salmerón-Ruiz M. A., Hernández P. J. R., Real-López M. (2025). Impact of screen and social media use on mental health. Anales de Pediatría (English Edition).

[B26-behavsci-16-00451] Kasturiratna K. S., Hartanto A. (2025). The moderating role of trait online disinhibition in exacerbating the online victim-bully cycle. Journal of Technology in Behavioral Science.

[B27-behavsci-16-00451] Kids Online (2022). Kids online Chile 2022: La relación de niños, niñas y adolescentes con el mundo digital.

[B28-behavsci-16-00451] Li Y. J., Cheung C. M., Shen X. L., Lee M. K. (2022). When socialization goes wrong: Understanding the we-intention to participate in collective trolling in virtual communities. Journal of the Association for Information Systems.

[B29-behavsci-16-00451] Liao J., Sun X., Fang X., Li F., Liu G., Lei L. (2024). How is parental phubbing associated with online trolling among early adolescents? The mediating role of alexithymia and the moderating role of online disinhibition. Current Psychology.

[B30-behavsci-16-00451] Liu M., Wu B., Li F., Wang X., Geng F. (2022). Does mindfulness reduce trolling? The relationship between trait mindfulness and online trolling: The mediating role of anger rumination and the moderating role of online disinhibition. Journal of Affective Disorders.

[B31-behavsci-16-00451] Mao Y., Hu B. (2025). From trolling victimization to reactive trolling: Moderated mediation effects of online disinhibition and motivations. Social Science Computer Review.

[B32-behavsci-16-00451] Márquez I., Lanzeni D., Masanet M. J. (2023). Teenagers as curators: Digitally mediated curation of the self on Instagram. Journal of Youth Studies.

[B33-behavsci-16-00451] Montez D., Kim D. H. (2025). How do silent trolls become overt trolls? Fear of punishment and online disinhibition moderate the trolling path. Social Media+ Society.

[B34-behavsci-16-00451] Moor L., Anderson J. R. (2019). A systematic literature review of the relationship between dark personality traits and antisocial online behaviours. Personality and Individual Differences.

[B35-behavsci-16-00451] Morales P., Urosa B., Blanco Á. (2003). Construcción de escalas de actitudes tipo likert.

[B36-behavsci-16-00451] Mueller-Coyne J., Voss C., Turner K. (2022). The impact of loneliness on the six dimensions of online disinhibition. Computers in Human Behavior Reports.

[B37-behavsci-16-00451] Muthén L., Muthén B. (2017). Mplus user’s guide.

[B38-behavsci-16-00451] Nagar K., Patidar A. K., Mudgal S. K., Gaur R., Patidar V. (2025). Relationship of digital game addiction with aggression and anger in the post COVID-19 era: A systematic review and meta-analysis. Investigación y Educación en Enfermería.

[B39-behavsci-16-00451] Nagata J. M., Memon Z., Talebloo J., Li K., Low P., Shao I. Y., Ganson K. T., Testa A., He J., Brindis C. D., Baker F. C. (2025). Prevalence and patterns of social media use in early adolescents. Academic Pediatrics.

[B40-behavsci-16-00451] Neira P., Zozaya L., Sádaba C., Feijoo-Fernández B. (2025). Percepciones de adolescentes españoles sobre la fiabilidad de la información en redes sociales. Revista OBETS.

[B41-behavsci-16-00451] Norambuena-Paredes I., Polanco-Levicán K., Tereucán-Angulo J., Sepúlveda-Maldonado J., Gálvez-Nieto J. L., Tavera-Cuellar C., Pérez-Ramírez S., Álvarez-Violante C., López-Tarango R. (2025). Factorial invariance of the Satisfaction with Life Scale (SWLS) in Mexican and Colombian university students. Behavioral Sciences.

[B42-behavsci-16-00451] Pérez-Torres V. (2024). Social media: A digital social mirror for identity development during adolescence. Current Psychology.

[B43-behavsci-16-00451] Polanco-Levicán K., Galvez-Nieto J. L. (2024). Psychometric properties of the Measure of Online Disinhibition (MOD) in Chilean university students. Current Psychology.

[B44-behavsci-16-00451] Polanco-Levicán K., Gálvez-Nieto J. L., Salvo-Garrido S., Norambuena-Paredes I., Vera-Gajardo N. (2025). Psychometric properties of the Identity Bubble Reinforcement Scale (IBRS) in a sample of Chilean adolescent students. Children.

[B45-behavsci-16-00451] Ray G., McDermott C. D., Nicho M. (2024). Cyberbullying on social media: Definitions, prevalence, and impact challenges. Journal of Cybersecurity.

[B46-behavsci-16-00451] Roberts J. A., David M. E. (2023). On the outside looking in: Social media intensity, social connection, and user well-being: The moderating role of passive social media use. Canadian Journal of Behavioural Science/Revue Canadienne des Sciences du Comportement.

[B47-behavsci-16-00451] Salza G., Samuel R. (2025). Digital engagement and youth: A scoping review of opportunities, risks, and the role of socioeconomic resources. Information, Communication & Society.

[B48-behavsci-16-00451] Sandoval M. S., de Casas Moreno P., Rojas-Estrada E. G. (2025). Instagram y su influencia en la salud mental de los jóvenes extremeños. Revista Española de Comunicación en Salud.

[B49-behavsci-16-00451] Schmitt H. S., Sindermann C., Montag C. (2025). Toxic disinhibition makes a versatile rulebreaker in cyberspace: Mapping online deviance with exploratory graph analysis and structural equation modeling. Acta Psychologica.

[B50-behavsci-16-00451] Scott J. E., Barlett C. P. (2023). Understanding cyber-racism perpetration within the broader context of cyberbullying theory: A theoretical integration. Children.

[B51-behavsci-16-00451] Scott R. A., Stuart J., Barber B. L. (2022a). Connecting with close friends online: A qualitative analysis of young adults’ perceptions of online and offline social interactions with friends. Computers in Human Behavior Reports.

[B52-behavsci-16-00451] Scott R. A., Stuart J., Barber B. L. (2022b). What predicts online disinhibition? Examining perceptions of protection and control online and the moderating role of social anxiety. Cyberpsychology, Behavior, and Social Networking.

[B53-behavsci-16-00451] Stevic A. (2024). Under pressure? Longitudinal relationships between different types of social media use, digital pressure, and life satisfaction. Social Media+ Society.

[B54-behavsci-16-00451] Stuart J., Scott R. (2021). The Measure of Online Disinhibition (MOD): Assessing perceptions of reductions in restraint in the online environment. Computers in Human Behavior.

[B55-behavsci-16-00451] Suler J. (2004). The online disinhibition effect. CyberPsychology & Behavior.

[B56-behavsci-16-00451] Syrjämäki A. H., Ilves M., Olsson T., Kiskola J., Isokoski P., Rantasila A., Bente G., Surakka V. (2024). Online disinhibition mediates the relationship between emotion regulation difficulties and uncivil communication. Scientific Reports.

[B57-behavsci-16-00451] Tan C. S. L. (2024). All by myself: Examining social media’s effect on social withdrawal and the mediating roles of moral disengagement and cyberaggression. European Journal of Marketing.

[B58-behavsci-16-00451] Tennakoon H., Betts L., Chandrakumara A., Saridakis G., Hand C. (2024). Exploring the effects of personal and situational factors on cyber aggression. Cyberpsychology: Journal of Psychosocial Research on Cyberspace.

[B59-behavsci-16-00451] Tian J., Zhu M., Zhao X. (2024). Why does sadism troll? The role of negative emotional reactions from others. Journal of Research in Personality.

[B60-behavsci-16-00451] Torrubia-Pérez E., Piñol-Piñol D., Segura-Meix M., Casanova-Garrigós G. (2025). Evaluación del impacto de los medios sociales sobre la salud de los y las adolescentes. Aposta: Revista de Ciencias Sociales.

[B61-behavsci-16-00451] Trizano-Hermosilla I., Gálvez-Nieto J., Alvarado J., Saiz J., Salvo-Garrido S. (2021). Reliability estimation in multidimensional scales: Comparing the bias of six estimators in measures with a bifactor structure. Frontiers in Psychology.

[B62-behavsci-16-00451] Vandenberg R. J., Lance C. E. (2000). A review and synthesis of the measurement invariance literature: Suggestions, practices, and recommendations for organizational research. Organizational Research Methods.

[B63-behavsci-16-00451] van der Wal A., Valkenburg P. M., van Driel I. I. (2024). In their own words: How adolescents use social media and how it affects them. Social Media + Society.

[B64-behavsci-16-00451] Voggeser B. J., Singh R. K., Göritz A. S. (2018). Self-control in online discussions: Disinhibited online behavior as a failure to recognize social cues. Frontiers in Psychology.

[B65-behavsci-16-00451] Wang L., Jiang S., Zhou Z., Fei W., Wang W. (2024). Online disinhibition and adolescent cyberbullying: A systematic review. Children and Youth Services Review.

[B66-behavsci-16-00451] Wang X., Qiao Y., Li W., Dong W. (2022). How is online disinhibition related to adolescents’ cyberbullying perpetration? Empathy and gender as moderators. The Journal of Early Adolescence.

[B67-behavsci-16-00451] Weisskirch R. S. (2025). Social media made me do it: Perceptions of social media influence, risky behaviors, and mental health among adolescents. Social Science Computer Review.

[B68-behavsci-16-00451] Wen R., Miura A. (2024). Development of the multi-dimensional Measure of Online Disinhibition, and examination of its validity and reliability. Osaka Human Sciences.

[B69-behavsci-16-00451] Yang J., Wang N., Gao L., Wang X. (2021). Deviant peer affiliation and adolescents’ cyberbullying perpetration: Online disinhibition and perceived social support as moderators. Children and Youth Services Review.

[B70-behavsci-16-00451] Ziegel L., Sjöland C. F., Zuo X., Fine S. L., Ekström A. M. (2025). Adolescent mental health and digital communication: Perspectives from 11 countries. Journal of Adolescent Health.

