# Supplementary material for: Psychometric Properties of the Measure of Online Disinhibition (MOD) in Chilean Adolescents
Source: Behav Sci (Basel). 2026 Mar 19;16(3):451. doi: 10.3390/bs16030451 (PMC13023658; doi:10.3390/bs16030451)
Supplement: Supplementary file 1 [file behavsci-16-00451-s001.zip › behavsci-4123429-Supplementary Materials.pdf]

Material suplementario / Supplementary Material

Se proporciona el siguiente material suplementario para acompañar nuestro manuscrito/ The following supplementary material is provided to accompany our manuscript:

Table S1. Escala de Desinhibición en Línea (Scale The Measure of Online Disinhibition, MOD):

Instrucciones/ **Instructions:**

A continuación, encontrarás cinco afirmaciones con las que puedes estar totalmente de acuerdo o totalmente en desacuerdo. Indica tu grado de acuerdo con cada afirmación / **Below, you will find five statements with which you may either totally agree or totally disagree. Please indicate your level of agreement with each statement.**

Versión original en español / Original version in Spanish

		Totalmente en desacuerdo	En desacuerdo	Indiferente	De acuerdo	Totalmente de acuerdo
1	Actúo de manera diferente en línea que fuera de línea	☐	☐	☐	☐	☐
2	Actúo más duro/rudo en Internet que en persona	☐	☐	☐	☐	☐
3	Soy menos cauteloso/a con lo que digo en línea que con lo que digo en persona	☐	☐	☐	☐	☐
4	Soy más asertivo/claro en línea que fuera de línea	☐	☐	☐	☐	☐
5	Soy más competitivo/a en línea que fuera de línea	☐	☐	☐	☐	☐
6	Tengo más confianza en línea que fuera de línea	☐	☐	☐	☐	☐
7	Soy más extrovertido/a en línea que fuera de línea	☐	☐	☐	☐	☐
8	Soy más capaz de discutir temas controversiales en línea que en persona	☐	☐	☐	☐	☐
9	Me resulta más fácil comunicarme con los demás en Internet que en persona	☐	☐	☐	☐	☐

10	Hago amigos/as más fácilmente en línea que fuera de línea					
11	Digo cosas en Internet que no diría en persona					
12	Mis comportamientos en línea están menos restringidos que en persona					

Versión en inglés / English version

		Strongly Disagree	Disagree	Indifferent	Agree	Strongly Agree
1	I act differently online than I do offline	☐	☐	☐	☐	☐
2	I act tougher on the internet than I do face-to-face	☐	☐	☐	☐	☐
3	I am less cautious about what I say online than about what I say in person	☐	☐	☐	☐	☐
4	I am more assertive online than I am offline	☐	☐	☐	☐	☐
5	I am more competitive online than I am offline	☐	☐	☐	☐	☐
6	I am more confident online than I am offline	☐	☐	☐	☐	☐
7	I am more outgoing online than I am offline	☐	☐	☐	☐	☐
8	I am more able to discuss controversial issues online than I am in person	☐	☐	☐	☐	☐

9	I find communicating with others easier on the internet than in person	☐	☐	☐	☐	☐
10	I make friends more easily online than I do offline	☐	☐	☐	☐	☐
11	I say things on the internet that I would not say in person	☐	☐	☐	☐	☐
12	My behaviours online are less restricted than in person	☐	☐	☐	☐	☐
